# Sensory signals of unloading in insects are tuned to distinguish leg slipping from load variations in gait: experimental and modeling studies

**DOI:** 10.1152/jn.00285.2022

**Published:** 2022-08-31

**Authors:** Christian M. Harris, Nicholas S. Szczecinski, Ansgar Büschges, Sasha N. Zill

**Affiliations:** ^1^Department of Biomedical Sciences, Joan C. Edwards School of Medicine, Marshall University, Huntington, West Virginia; ^2^Department of Mechanical and Aerospace Engineering, Benjamin M. Statler College of Engineering and Mineral Resources, West Virginia University, Morgantown, West Virginia; ^3^Department of Animal Physiology, Institute of Zoology, University, of Cologne, Cologne, Germany

**Keywords:** posture, sensory encoding, unloading, walking

## Abstract

In control of walking, sensory signals of decreasing forces are used to regulate leg lifting in initiation of swing and to detect loss of substrate grip (leg slipping). We used extracellular recordings in two insect species to characterize and model responses to force decrements of tibial campaniform sensilla, receptors that detect forces as cuticular strains. Discharges to decreasing forces did not occur upon direct stimulation of the sites of mechanotransduction (cuticular caps) but were readily elicited by bending forces applied to the leg. Responses to bending force decreases were phasic but had rate sensitivities similar to discharges elicited by force increases in the opposite direction. Application of stimuli of equivalent amplitude at different offset levels showed that discharges were strongly dependent upon the tonic level of loading: firing was maximal to complete unloading of the leg but substantially decreased or eliminated by sustained loads. The contribution of cuticle properties to sensory responses was also evaluated: discharges to force increases showed decreased adaptation when mechanical stress relaxation was minimized; firing to force decreases could be related to viscoelastic “creep” in the cuticle. Discharges to force decrements apparently occur due to cuticle viscoelasticity that generates transient strains similar to bending in the opposite direction. Tuning of sensory responses through cuticular and membrane properties effectively distinguishes loss of substrate grip/complete unloading from force variations due to gait in walking. We have successfully reproduced these properties in a mathematical model of the receptors. Sensors with similar tuning could fulfil these functions in legs of walking machines.

**NEW & NOTEWORTHY** Decreases in loading of legs are important in the regulation of posture and walking in both vertebrates and invertebrates. Recordings of activities of tibial campaniform sensilla, which encode forces in insects, showed that their responses are specifically tuned to detect force decreases at the end of the stance phase of walking or when a leg slips. These results have been reproduced in a mathematical model of the receptors and also have potential applications in robotics.

## Graphical abstract

**Figure:**
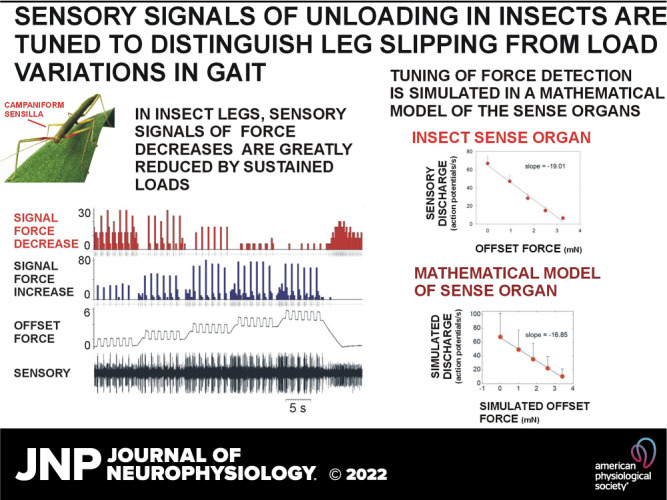

## INTRODUCTION

Decreases in load can have significant effects in the control of posture and walking (1–3). Previous studies have shown that motor responses elicited by leg unloading are not immutable but vary according to the context and temporal sequence of behaviors (4). Large decreases in load occur near the end of the stance phase of walking, before leg lifting in swing (5, 6). Sensory signals of unloading at the end of stance can inhibit leg muscles that generate support of body load and, concurrent with discharges of afferents indicating extension of limb joints, can aid in the activation of muscles that produce leg lifting and initiation of a step. “Stepping” reactions can also be elicited by perturbations that produce sudden leg unloading in standing and walking (7). These responses can be elicited in walking by loss of substrate contact (8) or perturbations of posture that shift the center of mass outside the base of support (9).

In contrast, load decreases imposed earlier in the stance phase can modulate activities of leg muscles that generate support and propulsion, rather than inducing leg lifting (10, 11). Load decreases occur in some legs when posture is perturbed by small displacements of the substrate (12). The activities of specific groups of leg muscles are strongly correlated with the extent and timing of the load decrement. In humans, imposing small changes in the ankle joint angle (at a rate and amplitude that did not elicit stretch reflexes), mimicked decreases in load that can occur in normal walking in the absence of large perturbations and produced decreased firing of the soleus muscle, an ankle extensor active in support and propulsion (13). These experiments suggest that the nervous system may be able to utilize the rate and magnitude of unloading in the decision to modulate muscle contractions versus initiate stepping. However, the specific sense organs that signal load decrements and that could mediate these motor reactions have not been determined (14). In vertebrates, load variations could alter discharges of Golgi tendon organs (11), muscle spindles (13), and receptors of the sole of the foot (15).

We have examined the effects of changes in load upon activities of the tibial campaniform sensilla, receptors that actively detect force increases and decreases via strains in the exoskeleton of the legs of insects (16–19). In both cockroaches and stick insects, the tibial sensilla are arranged in two subgroups with opposite sensitivities to the direction of bending forces (17, 20–22). Increasing bending forces upon a leg produces activation of one subgroup whereas load decreases elicit firing of the subgroup with opposite directional sensitivity (23). Alternating bursts of the subgroups occurs in walking of freely moving or partially restrained animals when legs are first loaded then unloaded in the stance phase (3, 24).

Previous studies also suggested that discharges to unloading were affected by the level of load and were not elicited by force decrements in the presence of large tonic loads (17). However, the specific ranges of sensitivities to force decreases and the parameters they encode have not been strictly determined. In the present study, we have systematically characterized responses of receptors to load increases and decreases at different load offsets. Data from these experiments support the idea that the receptors are specifically tuned to detect large or rapid force decreases in ranges close to complete unloading and can aid in eliciting motor responses depending upon the context and temporal sequence of the behavior. Furthermore, we have begun studying the mechanisms underlying sensory encoding of force decreases and shown that discharge frequencies can be correlated with viscoelastic properties (creep) of the leg cuticle. These data have also been utilized in tests of a mathematical model of campaniform sensilla (25). Similar response characteristics to leg unloading were obtained in the model in simulations using experimental data obtained in this study.

## METHODS

### Sensory Recordings

**Figure 1.**
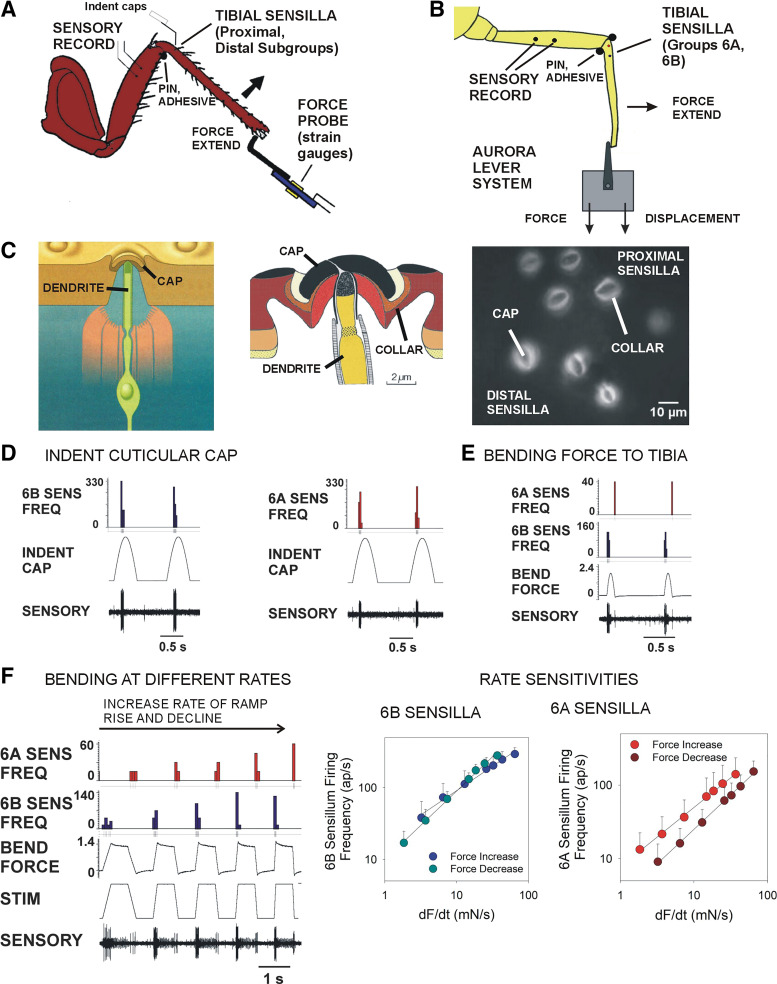
Preparations for recording discharges of tibial campaniform sensilla and responses to cap indentation vs. leg bending. *A* and *B:* recording preparations—activities of tibial campaniform sensilla were recorded through a pair of wires (placed adjacent to a nerve containing the receptor axons. In most experiments, forces were applied to the end of the tibia with a probe (containing strain gauges that was driven by a motor (*A*). In studies in stick insects, forces were also applied with an Aurora puller (*B*), which permitted independent control of force or displacement. *C*: structure of campaniform sensilla—(*left*): a campaniform sensillum consists of a sensory neuron whose dendrite inserts into a cuticular cap at the surface of the exoskeleton. Detailed drawing of cuticular cap [*middle*, after Gnatzy et al. (26)]. The cap of the campaniform sensillum is linked to the cuticle via a collar. Confocal fluorescence micrograph of cockroach tibial sensilla (*right*): the collars appear as bright rings of elastic cuticle surround the darkened (sclerotized) caps. *D*: indentation of cuticular caps—indenting the caps of individual stick insect tibial campaniform sensilla with a fine probe (time indicated by indent cap) produced discharges to force increases in 6B (*left*) and 6A (*right*) receptors but no firing to decreases in indentation force. *E*: brief application of bending forces (forced extension of the tibia) elicited firing of 6B sensilla to force increases and action potentials in 6A receptors to force decreases. *F*: application of bending forces at different rates of rise and decline produced more prolonged firing of 6A receptors that reflected the rate of force decrease (*left*). Plots of sensory firing frequencies vs. rate of force application show that similar rate sensitivities are found to decreasing forces and to increasing forces in the opposite direction (6A: *n* = 28, *N* = 2; 6B: *n* = 40, *N* = 4).

Experiments were performed on adult cockroaches (*Periplaneta americana* males obtained from Carolina Biological Supply) and stick insects [*Carausius morosus*, females obtained from colonies maintained at the Bielefeld University, the University of Cologne or from commercial suppliers (Backwater Reptiles)]. All animals were adult and selected to have firm exoskeletons (“al dente”) based upon squeezing the thorax. The methods for recording activities of tibial campaniform sensilla (Fig. 1, *A*–*C*) have been previously described (23, 24). Briefly, animals were first restrained on a resin-coated platform using staples and all nerves to the leg under study were severed in the thorax (Fig. 1, *A*and *B*). The leg was then positioned so that the plane of leg movement was parallel to the surface of the platform. The femoro-tibial joint was fixed using a pin and adhesive and the tarsus was amputated so that forces could be applied to the free end of the tibia. Small holes were made in the cuticle of the femur and fine silver wires (50 μm diameter) were inserted and positioned close to the nerves that contain the receptor axons (20, 23). Sensory activities were monitored during electrode placement, recorded using a custom-built amplifier (Michael Duebber, University of Cologne) and stored digitally via a Spike2 interface [Cambridge Electronic Design (CED), Cambridge, UK].

### Mechanical Stimulation

In most experiments, receptors were mechanically stimulated by applying forces to the tibial segment with movement resisted. Forces were imposed on the tibia using a probe mounted on a linear DC motor (Fig. 1*A*; Michael Dȕbbert, University of Cologne). In other studies, forces were generated using an Aurora Scientific actuator/transducer system (Aurora Scientific Instruments 300C) that permits characterization of mechanical properties by independent control and monitoring of force and displacement (Fig. 1*B*). Voltages to the motor or actuator were generated by the Spike2 interface [Cambridge Electronic Design (CED), Cambridge, UK]. The forces exerted upon the tibia by the motor were monitored with strain gauges attached to the probe. Signals of forces from all devices were calibrated using small weights.

### Data Analysis

Sensory units could be consistently distinguished by the amplitudes of extracellular action potentials and by selective ablation of sensilla subgroups (22, 26). The tibial sensilla in stick insects (Groups 6A and B) are spatially separated on the proximal tibia whereas in cockroaches the homologous subgroups (distal and proximal sensilla) are more adjacent in location (Fig. 1*C**, right*). As in our previous studies (27, 28), we selected and analyzed recordings in which sensory responses remained mechanically stable through lengthy sequences of stimulus repetitions at comparable force levels. Data on firing rates and forces were calculated using custom Spike 2 scripts. For plots, the rate of change of force was calculated directly from the recorded force (without filtering) in Excel software. Statistical tests were performed and plotted in SigmaPlot (Systat software) and SPSS (IBM; RRID:SCR_002865).

### Morphology

For study by confocal microscopy, cuticle of the proximal tibia was isolated and placed in Conray (a radiopaque dye that can be used as a clearing agent for insect cuticle) (22, 29, 30). Specimens were imaged using a Leica TCS SP5 II microscope at the Marshall University Microscopy facility. Measurements of cuticular caps were made on projection images and optical sections using ImageJ (v.1.43 u, NIH; RRID:SCR_003070).

## RESULTS

### Structure of Campaniform Sensilla and Compliance in Sensory Mechanotransduction

Responses to decreasing forces could result from mechanical properties in elements of campaniform sensilla that mediate mechanotransduction. Campaniform sensilla detect strains via the insertion of their dendrites to cuticular caps at the surface of the exoskeleton (Fig. 1*C*). The caps are linked to the surrounding exoskeleton by thin cuticular collars (Fig. 1*C*, *middle*). Recent studies (31, 32) have shown that the cuticular compliance (elasticity) may be reflected in gradients of fluorescence that can be visualized by confocal microscopy after image color separation (splitting color channels). Figure 1*C* (*right*) contains a confocal image (blue channel after color split) of the tibial sensilla (Group 6) from a hindleg of a cockroach: the cuticular caps appear as dark ovals surrounded by rings that fluoresce brightly. In addition, regions adjacent to the sensilla also fluoresce although less intensely. These findings support the idea that mechanical coupling of the rigid cuticular cap is via compliant and elastic collars that are linked to surrounding exoskeleton, which can show gradients of elasticity/rigidity (33, 34).

### Responses to Decreasing Forces Occur to Leg Bending Not to Direct Cap Indentation

Responses of tibial campaniform sensilla of stick insects and cockroaches were tested to both indentation of the cuticular caps and to bending of the tibia. Figure 1*D* shows recordings in which the caps of 6B (*left*) and 6A (*right*) sensilla of stick insects were indented using a fine probe. In each subgroup, discharges of the receptors were limited to the time of force increase in cap indentation not to the decline and no discharges of 6A sensilla occurred when 6B caps were stimulated. Similar tests in cockroaches showed that indentation of proximal or distal tibial receptors (Fig. 1*C*, *right*) did not produce spikes in other receptors, despite their close proximity (35, 36). In contrast, reciprocal firing of the subgroups occurred when bending forces were applied to the tibia (Fig. 1*E*). Recordings in which forces were applied using ramp and hold functions of different rates of rise and decline (Fig. 1*F*, *left*) showed that the discharges to force declines consistently encoded the rate of force. Similar sensitivities to the rate of change of force are seen both in responses to force increases and in encoding of force decreases (Fig. 1*F*, *right*; 6A, *n* = 28, *N* = 2; 6B, *n* = 40, *N* = 4).

### Effects of Forces Applied at Different Baseline Levels as Staircase Functions

**Figure 2.**
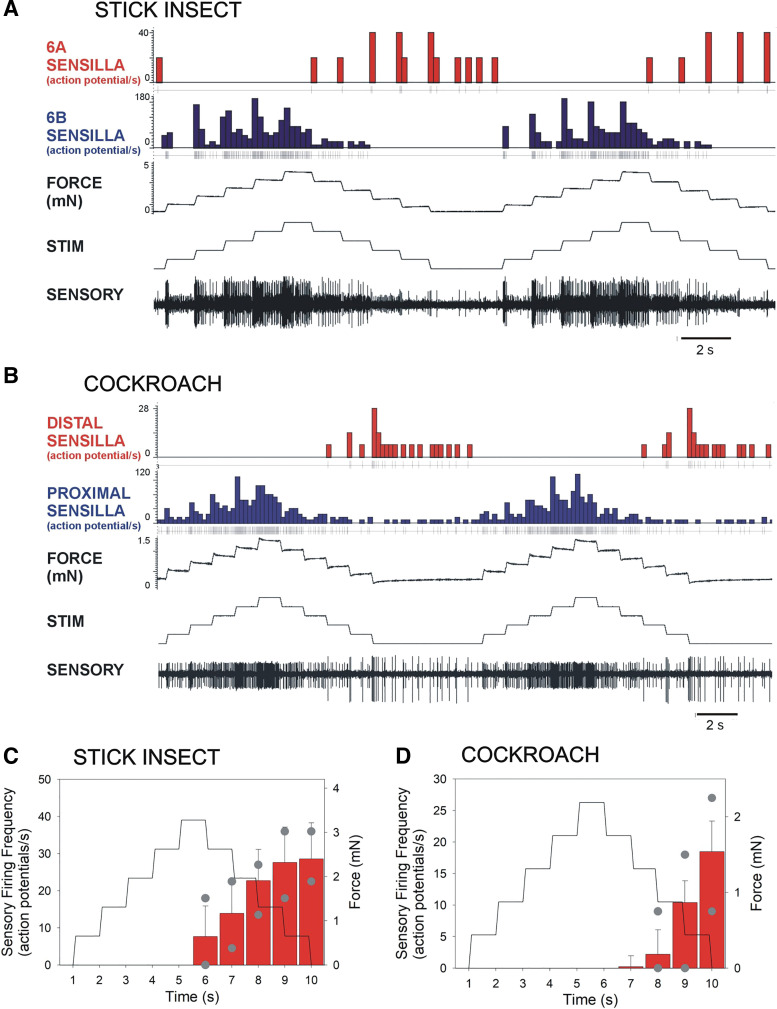
Responses of sensilla to bending forces applied as “staircase” functions at progressively increasing and decreasing levels. *A* and *B*: bending forces were applied to the tibia in the direction of extension (movement resisted) as step increases and decreases (STIM, stimulus) in a stick insect (*A*) and cockroach (*B*). In stick insects, 6B receptors discharged to force increases while 6A receptors respond to the step decreases. In cockroaches, a similar pattern of firing to force increases and decreases was obtained from proximal and distal tibial campaniform sensilla. In both animals, firing was phasic to step decreases but more prolonged when applied forces declined to their initial values. *C* and *D*: plots of sensilla discharges to force decreases in response to stair case functions (gray dots indicate maximum and minimum values). In both stick insects and cockroaches, discharges of receptors to force decreases were maximal as force levels approached zero. The range of responses was broader in stick insects than in cockroaches (*C*: *n* = 137; *N* = 3; *D*: *n* = 56, *N* = 3).

To quantify discharges to force decreases and compare responses of stick insect and cockroach tibial campaniform sensilla, we utilized a staircase function waveform that first increased to a maximum level in five steps of the same amplitude and rate and then declined in equivalent steps (Fig. 2). In each species, one subgroup (stick insect 6B receptors, cockroach proximal sensilla) fired at increasing levels to the force increases whereas the opposite subgroup (stick insect 6A sensilla, cockroach distal receptors) discharged to force decrements (Fig. 2, *A*and *B*). In stick insects, discharges regularly consisted of units of large (6B large receptors) and smaller extracellular amplitude (22). Plots of the mean discharges during the ramp decrease (Fig. 2, *C* and *D*) showed that, in both animals, discharges to force decrements were minimal or absent at high levels of load but vigorous phasic firing occurred when load was completely reduced (confirming previous findings) (17, 22). These tests also demonstrated that the effect of sustained loading was more pronounced in cockroach tibial sensilla that showed a narrower range of responses than in stick insects (cockroach *n* = 135, *N* = 3; stick insect *n* = 48, *N* = 3).

### Effects of Offset Loads on Discharges to Equivalent Forces

**Figure 3.**
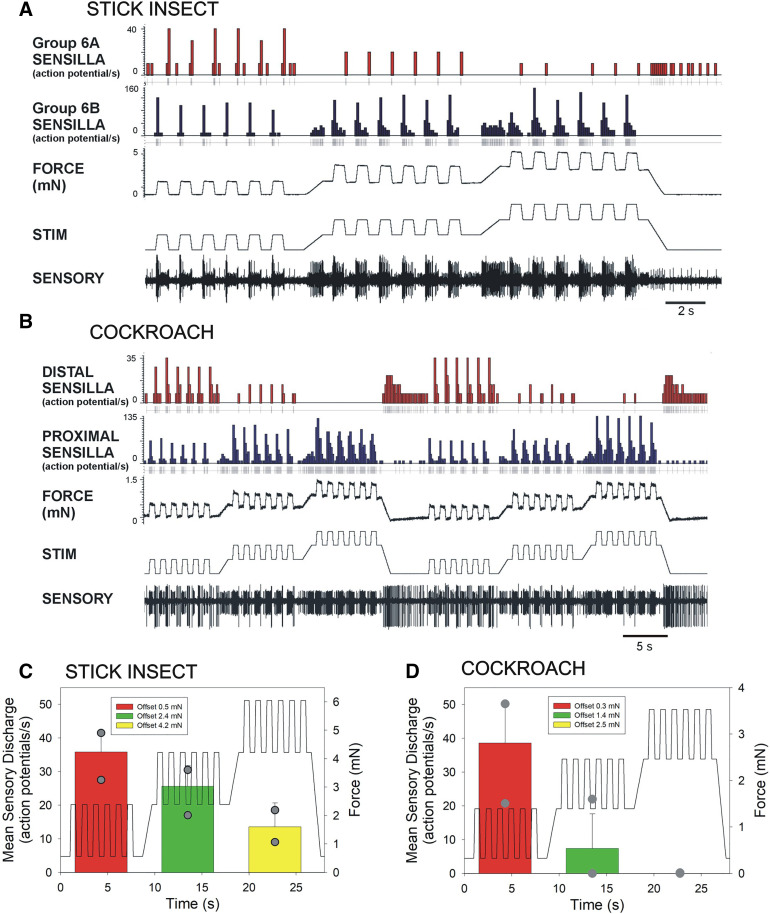
Sensory responses to forces applied as ramp and hold functions at different offset levels. *A* and *B*: responses of tibial sensilla of a stick insect (*A*) and a cockroach (*B*) to application of forces as the same ramp and hold functions (direction of joint extension) at different offset levels. Discharges of stick insect 6B sensilla and cockroach proximal receptors increased at higher offset levels. Firing to force decreases was strongly dependent upon the level of load: discharges were minimal/did not occur after large load offsets but were intense and prolonged when at loads were decreased to the initial offset value. *C* and *D*: plots of mean discharges to force decreases (gray dots indicate maximum and minimum values; see text for discussion) (*C*: *n* = 336, *N* = 4; *D*: *n* = 506; *N* = 3). STIM, mechanical stimulus.

To reduce the potential effects of long-term adaption to sustained loads that could occur in forces applied as staircase functions, responses of the receptors were systematically tested using application of ramp and hold functions of the same rate and amplitude at different levels of loading applied as offsets (Fig. 3). Large sensilla of subgroups of receptors that encode force increments in the direction of joint extension (stick insect Group 6B, cockroach proximal sensilla) fired vigorously during period of ramp rise force increases applied with minimal offset and showed more prolonged tonic discharges to loads from higher baseline levels. However, receptors that encoded force decreases (6A and distal sensilla) were strongly affected by changes in load offset, even though the rate and magnitude of force decline was equivalent in all tests. Figure 3, *C*and *D* shows histograms of the mean sensory discharge rates during the period of force decrement, which decline dramatically when offset loads are applied. The response range in stick insects sensilla was broader and firing was more sustained than in cockroaches, although sensilla of both animals showed intense and prolonged discharges when forces returned to zero at the end of the offset waveform.

### Quantifying the Effects of Load Offset

**Figure 4.**
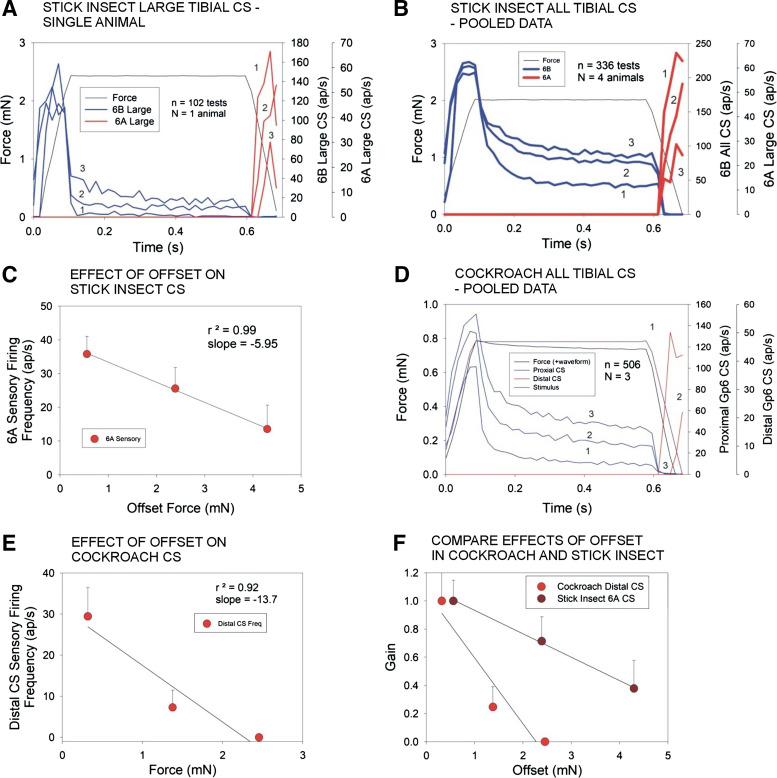
Effects of offset on force encoding in tibial sensilla campaniform sensilla (CS). *A*: line plot of mean discharge frequencies of stick insect large amplitude tibial campaniform sensilla (6B, blue and 6A, red) to forces applied to the tibia using ramp and hold waveforms at different offset levels (Offset = 1—0 mN, 2—2.9 mN, 3—5.4 mN) in a single preparation. Responses to force increases (6B receptors) rapidly adapted but were more sustained at higher offset levels. Sensillum discharges (6A sensilla) were highest at zero offset and decreased substantially at increasing offset levels. *B*: plot of discharges of all 6B sensilla. Firing is sustained during the hold phase when 6B smaller sensilla are included but only phasic in 6A receptors. *C*: plot of mean sensory firing during the period of decreasing forces in stick insect 6A receptors. *D*: line plot of mean discharges of cockroach tibial campaniform (proximal receptors, blue and distal sensilla, red) to forces applied to the tibia using ramp and hold waveforms at different offset levels (mean amplitude = 0.85 mN ± 0.42; Offset = 1—0 mN, 2—0.92 mN, 3—1.84 mN). Proximal sensilla fired vigorous and sustained discharges that reflected the magnitude of tonic offsets. Distal receptors showed intense phasic firing to force decreases and the firing frequency decreased and then ceased when progressively larger offsets were applied. *E*: plot of mean sensory firing during the period of decreasing forces in stick insect 6A receptors. *F*: plot of relative gain of sensory discharges (calculated as the ratio of firing frequencies to minimal offset) in both species. In cockroaches, the slope of the decline is greater than in stick insects, which may be correlated with differences in cuticle properties (same data set as Fig. 3).

To compare the characteristics of campaniform sensilla in different species, the specific effects of load offsets on the patterns of sensory discharges are further analyzed in Fig. 4. In stick insects, discharges of the large 6B receptors were phasic and limited to the ramp phase of force increase at lower offset levels and more sustained firing only occurred at higher force levels (Fig. 4*A*; offsets 0, 2.9, and 5.4 mN). The firing frequency during the ramp rise was similar at all offset levels and reflected the rate of change of force (see also Ref. 17). Plots that included both the large and more tonic small 6B sensilla showed graded increases that reflected the force offset (Fig. 4*B*) but saturation occurred at higher offset levels. Stick insect 6A sensilla were discharged during the period of ramp decline but the firing frequencies decreased as offset loads increased (Fig. 4*C*; slope −5.95). Figure 4, *D*and *E* shows similar plots for discharges of the tibial campaniform sensilla in cockroaches. Proximal tibial receptors fired intensely during the ramp rise and then adapted during the hold phase at all offset levels (Fig. 4*D*; Offsets 0.92, 1.84, and 2.45 mN). Discharges during the ramp phase were somewhat lower at smaller offsets but relatively constant at offset levels above 1.84 mN (see also Ref. 17). Increasing the offset level was clearly reflected in the tonic discharges of proximal receptors during the hold phase. Firing of distal receptors was, however, greatly reduced by relatively low offset forces and largely eliminated at higher levels (Fig. 4*E*, slope −13.7). The effects of load offsets on discharges to decreasing forces in both species are summarized and compared in the plots in Fig. 4*F*. Sensitivities (gain) of receptors detecting force decreases are reduced in stick insects and completely inhibited in cockroach when they occur during sustained loading.

### Evidence That Discharges to Decreasing Forces Depend upon Cuticle Viscoelastic Properties

**Figure 5.**
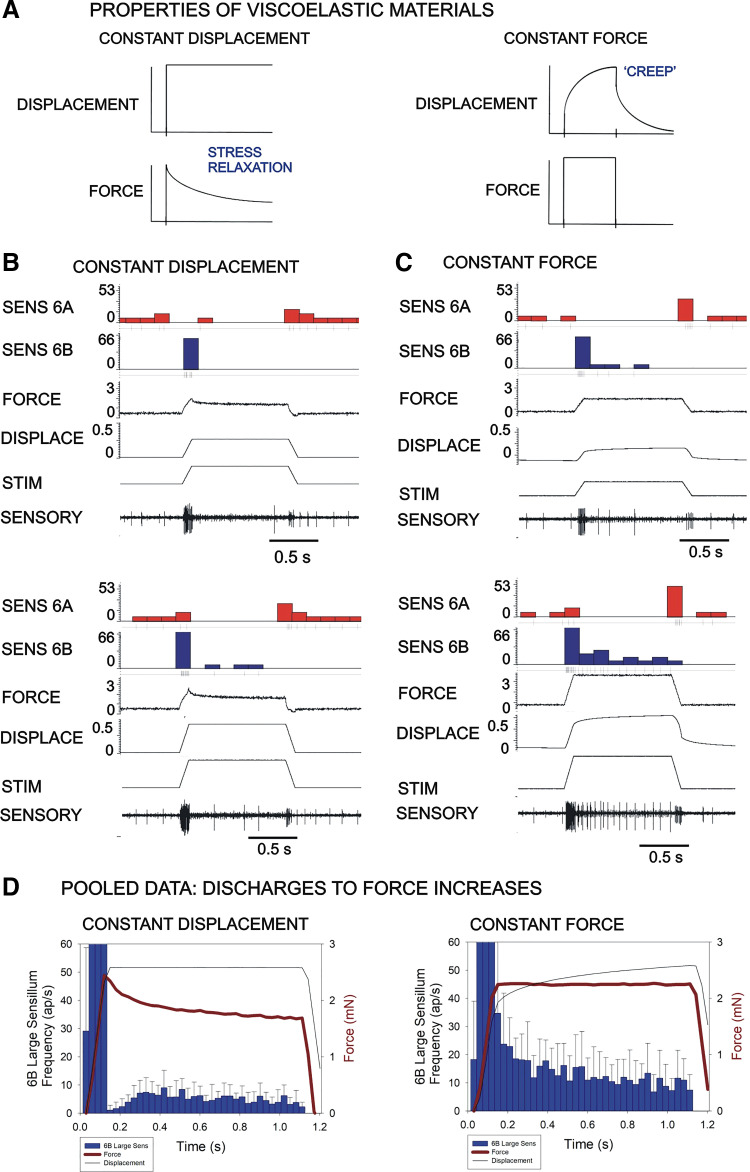
Force encoding and cuticle viscoelasticity. *A:* the Aurora system allows for independent control of force and displacement. In response to maintained displacement (*left*), viscoelastic materials exhibit “stress relaxation” and resisting forces decline gradually. When forces are held constant (*right*), “creep” occurs and displacement gradually increases. *B*: bending forces applied as constant displacements to the stick insect tibia elicited discharges of the 6B sensilla that declined rapidly after the onset of the hold phase due to stress relaxation. *C*: stimuli applied as constant forces produced firing large 6B receptors that was prolonged at low force levels. At moderate force levels, discharges were sustained during the entire hold phase. Measurements of displacement showed gradual creep in these tests. *D*: pooled data on effects of force increases—application of forces as constant displacements (*left*) produced a brief cessation of discharge at the start of the hold phase due to stress relaxation that did not occur when forces were applied as constant forces (*right*) (*D*: constant displacement: *n* = 137, *N* = 5; constant force: *n* = 119, *N* = 5; note the stimulus channel indicates the waveform applied to the motor so no absolute scale is included.). STIM, mechanical stimulus.

To further characterize the mechanisms underlying discharges to force decreases, we characterized responses of stick insect tibial sensilla to bending forces applied with an Aurora actuator/transducer system. This system allowed mechanical stimuli to be applied and measured as constant (determined) forces or displacements (Fig. 5*A*), permitting evaluation of material viscoelasticity. In constant displacement mode, the magnitude of displacement of the end of the tibia is held constant while forces are allowed to vary (similar to applying forces with the linear motor) and in viscoelastic materials can show stress relaxation (Fig. 5*A*, *left*). In constant force mode, the system maintains force levels by generating increasing displacements to compensate for viscoelastic “creep” (Fig. 5*A*, *right*).

Bending forces applied to the stick insect tibia showed stress relaxation (decline in force) in the cuticle to stimuli of constant displacement (Fig. 5*B*) and “creep” to waveforms applied at constant force (Fig. 5*C*). Discharges of campaniform sensilla to stimuli applied to the end of the tibia differed considerably in the two modes. Stimuli applied as constant displacements elicited discharges of the 6B sensilla that declined rapidly after the onset of the hold phase as forces decreased due to stress relaxation (Fig. 5*D*, *left*). Firing of 6B receptors was only maintained at low tonic levels to higher applied displacements. In contrast, in the constant force mode (which compensated for cuticle viscoelasticity by varying displacement), discharges of large 6B receptors were prolonged at low force levels and firing to moderate forces adapted to tonic levels that were sustained during the hold phase (Fig. 5*D*, *right*). Measurements of the displacement showed gradual increases reflecting the effects of viscoelastic creep in the cuticle. The magnitude of these effects varied in different animals depending upon the apparent of degree of sclerotization of the cuticle (“hard” versus “soft” animals) but differences seen in adaptation of sensory response were present, to some degree, in all preparations.

**Figure 6.**
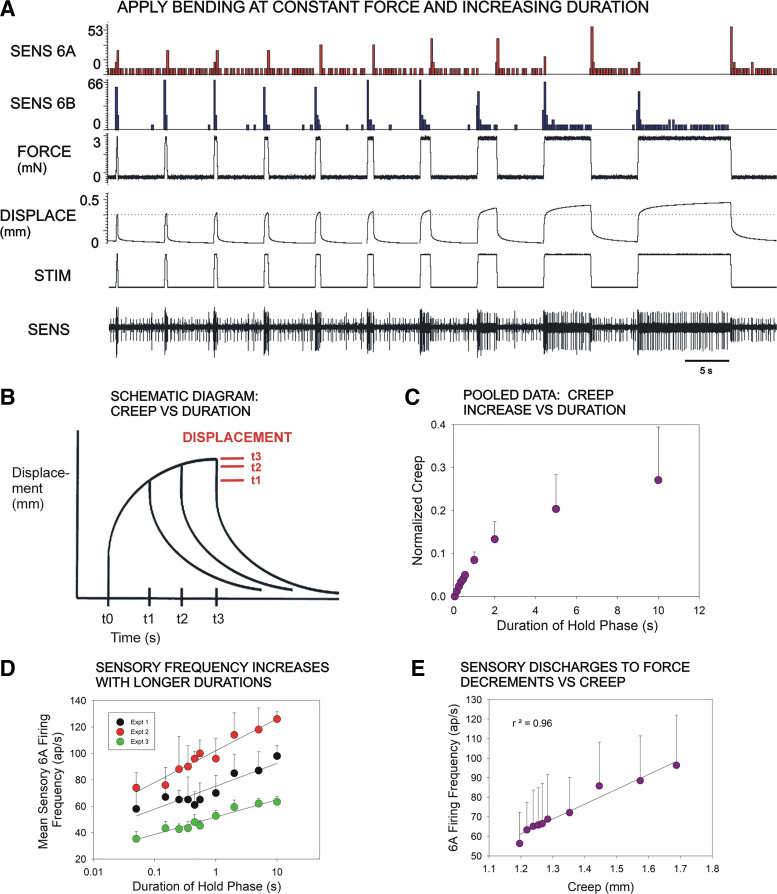
Responses to decreased forces and mechanical “creep.” *A*: recording of stick insect tibial sensilla to forces applied (in constant force feedback mode) to ramp and hold functions applied at the same force levels but with increasing durations of the hold phase. Forces are held at constant levels but displacements reflect viscoelasticity in the cuticle. Firing of 6B sensilla are sustained reflects at all durations of the hold phase but discharges of 6A receptors to force decreases increased following longer hold phases. *B*: diagram of effect of increasing duration on mechanical “creep”—creep (increase in displacement) gradually increases but reaches higher levels in longer displacements. *C*: plot of mean “creep” vs. duration in three preparations. *D*: plot of mean firing frequencies of 6A receptors to force decrease vs. duration of the preceding hold phase in three preparations (*experiments 1*, *2*, and *3*). The discharges frequencies of all receptors show similar duration dependent increases. *E*: discharges of 6A receptors vs. “creep”—the sensory firing frequency reflects the effects of cuticle viscoelasticity. (*C*: *n* = 42; *N* = 4; *D*: each test at 10 durations: *experiment 1*: *n* =14; *experiment 2*: *n* = 10; *experiment 3: n* = 12; total 36 tests; *E*: *n* = 46 test at 10 durations, *N* = 4). STIM, mechanical stimulus.

As responses to force decreases were apparently larger following more sustained displacements, we utilized the system to apply constant forces of varying duration to characterize time-dependent effects of the cuticle on sensory encoding. Figure 6*A* shows a recording of sensory discharges to forces applied (in constant force feedback mode) to ramp and hold functions applied at the same force levels but with increasing durations of the hold phase. Firing of the 6B sensilla to force increases is sustained at all durations of the hold phase. However, discharges of 6A receptors to the subsequent force decreases were significantly higher following longer hold phases, even though the magnitude of the force was held constant (Fig. 6, *A*and *D*). Measurements of displacements in these tests showed gradual increases in the hold phase that reached higher levels following longer displacements, reflective of time-dependent viscoelastic creep in the cuticle of the leg (Fig. 6, *B*and *C*). Plots of the sensory discharges to decreasing forces showed a strong correlation with magnitude of “creep” that occurred during the hold phase (Fig. 6*E*). These preliminary findings are consistent with the idea that discharges to decreasing forces are dependent, in part, upon viscoelastic properties of the cuticle.

### Mathematical Model of Campaniform Sensilla Reproduces Characteristics of Responses to Decreasing Forces

We have developed a mathematical model of force encoding by campaniform sensilla (25, 37). The model calculates the discharge frequency of the receptors as the sum of an adaptive function (instantaneous force minus low-pass filtered force) and a function reflecting tonic sensitivity (adjusted by an offset). Specifically, the discharge frequency, y, is a function of the instantaneous force applied to the tibia, u, and the low-pass filtered force, x:

*y* = max[0,*a*·(*u* − *x*) + *c*·*u* + *d*],

where *a* scales the adaptive term (*u* − *x*), *c* scales the tonic term *u*, and *d* is a constant offset. The low-pass filtered force variable *x* functions like a dynamic threshold and is calculated via the following differential equation:

$$\tau \cdot \frac{\text{d}x}{\text{d}t}=\text{sign}\left(u-x\right)\cdot {\left|u-x\right|}^{b}.$$

In short, *x* is driven to the value of *u* over time, with *x* changing more slowly as it approaches *u*. Although the dynamics of *x* could be approximated by many different functions, the form presented here was deliberately chosen to ensure the model produces key properties of CS (campaniform sensilla) responses, in particular, an emergent power law relationship between the rate of change of the stimulus force and the sensory discharge (25, 37), as reported in multiple prior experimental studies (23, 24, 38).

The constant τ describes the rate at which *x* approaches *u*. The constant *b* accounts for the nonlinear behavior of CS adaptation over time. Because none of the model parameters (e.g., *a*, *b*, *c*, and *d*) relate to specific mechanical or electrochemical processes, this model is descriptive, not mechanistic. Nevertheless, we will show that the adaptive behavior of the model is consistent with the adaptive mechanical behavior of CS, e.g., viscoelastic creep under sustained load.

**Figure 7.**
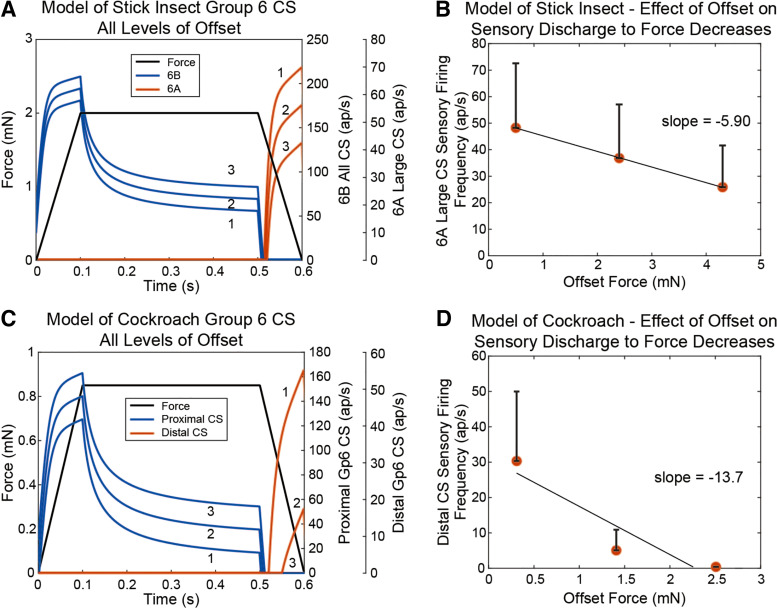
Simulation of experimental results on effects of offset loads in a mathematical model of the receptors. *A*: the model, tuned based on previous stick insect results, produces 6B responses that are relatively insensitive to the offset force but produces 6A responses that are relatively sensitive to the offset force. This results from the interplay between the adaptive function and tonic function in the model. Compare these results from experimental results in Fig. 4*B.* *B*: the model’s discharge during offsets is inversely proportional to the offset force, as seen in the experimental results in Fig. 4*C.* *C*: the model, tuned based on previous cockroach results, produces proximal campaniform sensilla (CS) responses that are relatively insensitive to the offset force but produces distal responses that are relatively sensitive to the offset force. Compare these results from experimental results in Fig. 4*D.* *D*: the model’s discharge during offsets is inversely proportional to the offset force, as seen in the experimental results in Fig. 4*E.*

Figure 7 show the results of simulated responses to forces imposed as ramp and hold functions of equivalent magnitude and rate at different offsets for the tibial campaniform of stick insects (Fig. 7*A*) and cockroaches (Fig. 7*C*). In both species, afferents (Fig. 7*A*, 6B sensilla; Fig. 7*C*, proximal receptors) show a phasic increase in discharge during the ramp that reflects the rate of force increase and a tonic discharge that is sustained in the hold phase. When forces decrease, the activities of 6B and proximal receptors cease immediately. Sensilla that encode force decreases (Fig. 7*A*, 6A sensilla; Fig. 7*C*, proximal tibial receptors) discharge to offsets close to zero but activities are decreased or firing ceases to larger offset loads. Figure 7, *B*and *D* shows plots of the model’s afferent firing frequencies of 6A sensilla in stick insects (Fig. 7*B*) and proximal receptors in cockroaches (Fig. 7*D*) at different simulated offset loads. As in the biological data (cf., Fig. 4, *E*and *F*), sensory discharges are maximal at offset values close to zero and decrease with larger offset values.

**Figure 8.**
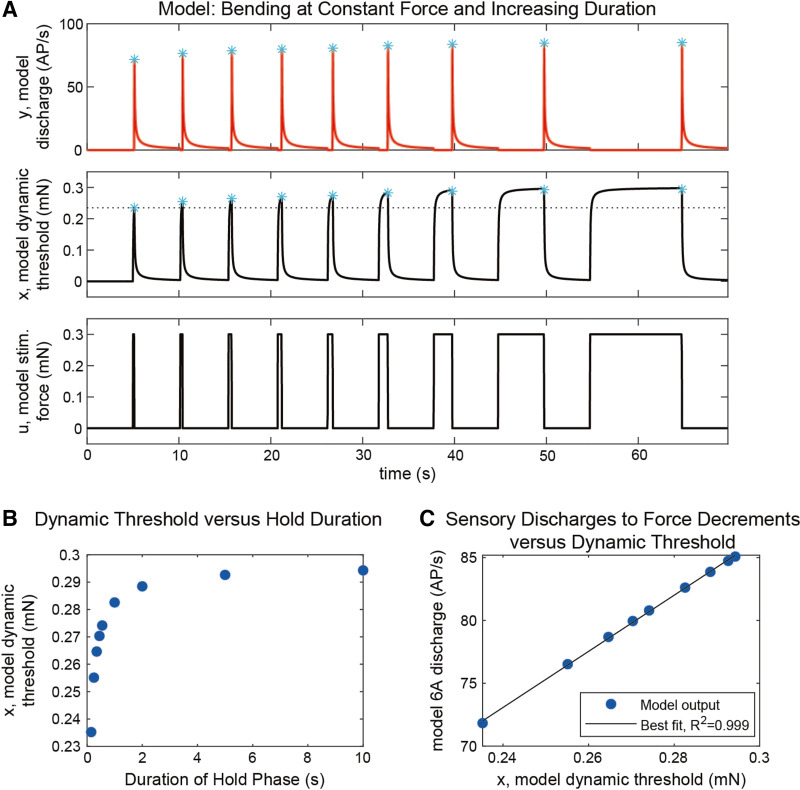
Simulation of experimental results on effects of stimulus duration/viscoelastic properties in a mathematical model of the receptors. *A*: the model campaniform sensilla (CS) responses to unloading (*top*) exhibit discharge amplitude that is proportional to the duration of the hold phase of the stimulus (cyan asterisks). Plotting the model’s dynamic threshold variable *x* (*middle*) in response to the stimulus force *u* (*bottom*) shows that *x* exhibits “creep” like that measured in the animal’s cuticle. The amount of “creep” in response to the first stimulus is indicated by the dotted horizontal line. The maximum creep is indicated by cyan asterisks. *B*: the maximum value of *x*’s “creep” depends on the duration of the hold phase of the stimulus (compare to Fig. 6*C*). *C*: the sensory discharge in response to force decrements is proportional to the amount of “creep” that *x* undergoes.

Figure 8 shows simulations of application of ramp and hold functions of constant amplitudes and rates of rise and decline at increasing durations. Figure 8*A* shows that the simulated CS fires bursts of action potentials when the force stimulus ends, as observed in stick insect group 6A CS (cf., Fig. 6*A*). Figure 8*A* also plots the applied force stimulus *u* and the model’s adaptive variable, *x*. As each force stimulus lengthens, the variable *x* progresses further from 0, its resting value. This transient behavior mimics the cuticle “creep” observed in animal experiments, suggesting that the model incidentally mimics the material properties of the animal. Figure 8*B* shows the values of *x* (dynamic threshold) at different durations of force application. The dynamic threshold increases at longer durations, similar to the values of creep in the cuticle (cf., Fig. 6*C*). Figure 8*C* is a plot of the simulated discharge of the 6A receptors versus the dynamic threshold. The firing frequency is related to the dynamic threshold, paralleling changes in creep in the cuticle. However, the afferent firing frequencies are not equivalent, as the value of the cuticle stiffness (elastic modulus), and thus the parameter values for the model, has not been determined for these data. These results show that the model replicates the effects of offset forces on sensory discharges. Simulations of viscoelastic creep can emerge from elements in the model, but further experiments are required.

### Experimental Confirmation of Model Predictions

**Figure 9.**
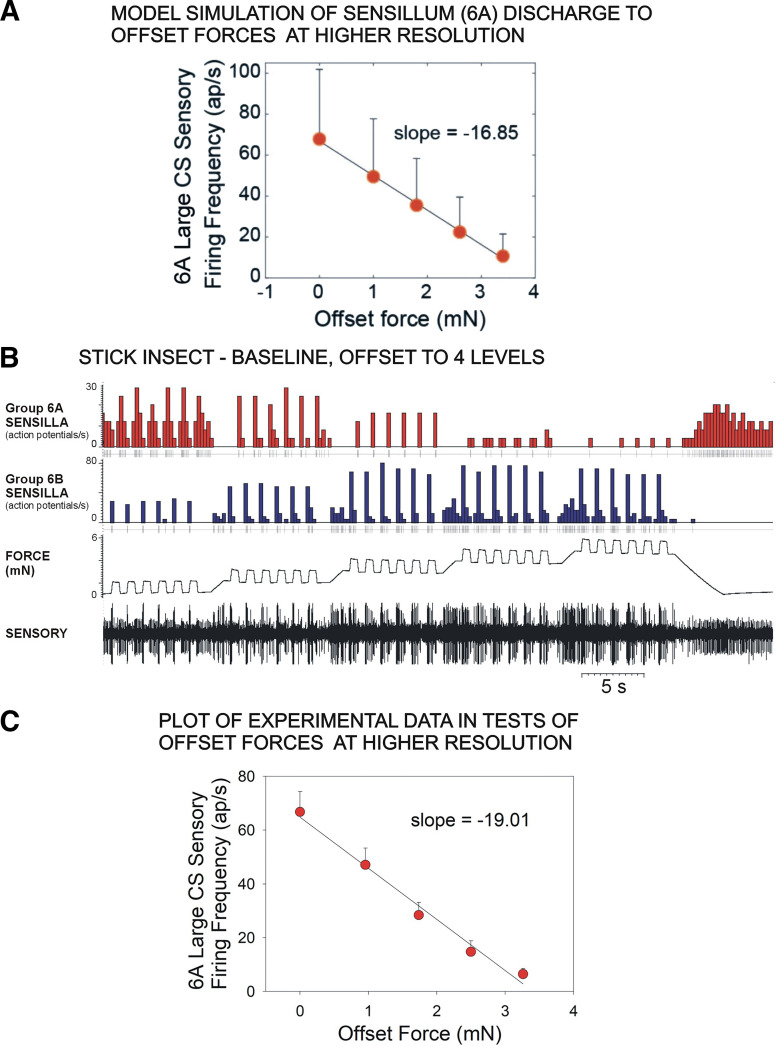
Predictive use of model: effects of force offset at higher resolution. *A:* model simulation of discharge of 6A sensilla to force decrements that would occur at offsets forces applied at five levels. *B*: experimental test of model—recording of stick insect tibial campaniform sensilla (CS) to forces applied as in the simulation. *C*: plot of discharges of 6A sensilla to offset forces applied as small steps. Sensory encoding is progressively decreased by increasing offset forces in both the model and experimental data (*C*: *n* = 1,500 tests, *N* = 3 animals).

The mathematical model was also used predictively to examine the effects of offset forces on sensory discharges at higher resolution. Figure 9*A* is plot of a simulation of the effects of offset forces applied in smaller steps on sensory discharges of stick insect group 6A sensilla to force decrements (similar to the plot in Fig. 7*C*). The model indicates that offset forces produce graded decreases in sensilla responses to progressive force decrements (slope = −16.85). We tested this experimentally by applying forces to the tibia of stick insects as a series of ramp hold increases and decreases at different offset levels (Fig. 9*B*), as in the experiment shown in Fig. 3*A* but with an increased number of offsets and a slightly larger range of forces. The pooled data from these tests is shown in Fig. 9*C*, which plots the discharges of 6A sensilla versus force at baseline and four incremental offsets (*n* = 50 sets of 6 repetitions at each level, total 1,500 tests, *N* = 3 animals). The experimental results are quite similar to the model predictions (*r*^2^ = 0.99, slope = −19.0), although the absolute firing frequencies (which depend upon the amplitude and rate of force decrease) varied.

## DISCUSSION

The major goals of the present study were to quantify and model responses of sense organs that detect decreasing forces, which are important in the initiation of the swing phase of walking and in generating compensatory steps following perturbations in the stance phase (39). Our data confirm previous studies and indicate that responses of tibial campaniform sensilla form a continuum, in which discharges are maximal when complete unloading occurs but minimal or absent when legs have large sustained loads. Tests of force application with ramp and hold functions of varying durations demonstrated that sensory discharge frequencies to force decrements were dependent upon viscoelasticity properties of the cuticle as they increased following longer “hold” phases. These data support the idea that discharges to force decreases result from time-dependent reversible deformations of the exoskeleton (creep). All sensory discharges are strongly rate sensitive supporting the idea that deformations in the cuticle may activate the same directionally sensitive mechanisms of mechanotransduction at the cuticular cap and sensory dendrite in both force increases and decreases. The discharges to force decreases are further tuned by mechanical/membrane properties to distinguish decreases in load that occur at the end of stance or following loss of substrate contact/support from load changes that occur during the stance phase due to variations in gait. Many of these findings have been successfully reproduced in a mathematical model of the receptors.

### Campaniform Sensilla of the Legs Do Not Show “On-Off” Responses

A number of sense organs show responses to both increases and decreases in applied forces, such as Pacinian corpuscles (40) and receptors of the skin of the foot (41). To determine the source of responses decreases in bending forces in campaniform sensilla, we systematically compared sensory discharges to direct mechanical stimulation of cuticular caps (site of mechanotransduction) and to bending of the tibia. Responses to force decreases did not occur in any tests of cap indentation and release in tibial sensilla of stick insects or cockroaches but were regularly elicited by bending forces applied to the leg (similar to findings of Ref. 42). Thus, sensilla of the legs do not show “on-off” responses to stimuli applied at the site of mechanotransduction. “On-off” responses were found in campaniform sensilla of the wings of flies (43, 44) to mechanical stimuli applied adjacent to the receptors, although responses of those receptors were unidirectional to direct electrical stimulation of the cuticular caps (43), suggesting that bidirectional responses result from mechanical properties of the cuticle of the wings.

### Leg Bending: Responses to Force Decreases Are Dynamic and History Dependent

Responses to decreasing forces were readily elicited in both stick insects and cockroaches to imposed bending of the tibia. Tests in which the rate of force decline was varied (Fig. 1, *E* and *F*) showed that sensory discharges in both species effectively encode the rate of change of force (−d*F*/d*t*), although firing was phasic and most often limited to the period of actual force decrement. However, sensory discharge to force decrements was more prolonged after force increments were applied for longer durations using staircase functions (Fig. 2) or in tests of stimuli applied at different offset levels (Fig. 3). These discharges showed adaptation qualitatively similar to that seen in responses to increases in bending forces applied in the opposite direction. These results support the view that the response characteristics to decreasing forces are similar to discharges to transient force increases in the reverse direction. Responses to decreasing forces are also seen in leg campaniform sensilla of other insects (45) and in mechanoreceptors of crustacea (46) but the specific parameters they encode have not been determined.

### Contribution of Viscoelastic Properties to Sensory Discharges: experimental Monitoring of Force is Essential

In the present study, we also generated forces with a system that can generate controlled forces or displacements, similar to devices used in earlier studies that examined transduction of mechanical forces and viscoelasticity in vertebrate cutaneous mechanoreceptors (47, 48). When forces are applied as constant (controlled) displacements they are resisted by elastic properties but, in viscoelastic materials, there is a gradual decline in force due to stress relaxation. When forces are held constant, displacements initially increase rapidly (due to elasticity) but viscoelastic materials show subsequent “creep” in displacement (changes in shape).

These properties were evident when forces were applied to the tibia. The tibia showed stress relaxation and decline in force level to ramp and hold functions of constant displacement (Fig. 6, *B* and *D*, *left*). The tibial sensilla showed brief cessation of firing at the start of the hold phase and marked adaptation. Sustained discharges could only be elicited at high force levels. Mechanical stimuli applied as constant forces (Fig. 6, *C*and *D*, right) elicited discharges of CS that showed prolonged firing and reduced adaptation even at moderate levels of force application. These discharges closely resembled the responses obtained from CS in freely standing cockroaches when the effect of body weight on the legs was increased by attaching a magnet to the thorax and generating magnetic fields by a coil below the arena (38). Thus, the constant force mode more closely resembled the effects of changes in load in intact animals.

It is important to note that the similar changes occurred when stimuli were applied repeatedly in successive tests. Future studies are planned to test and quantify the possible occurrence of longer term adaptation in the system but, at the moderate levels of force applied in the current system, the changes appear to be completely reversible, indicating that no permanent deformation had occurred (i.e., structural damage or failure).

It also should be noted that many studies in insects have only monitored displacement in evaluating sensory discharges and nervous system encoding of applied forces and loads (20, 21, 49). The present study has shown that effects of displacements can vary considerably depending upon cuticle properties. The consequent stress relaxation can potentially produce substantial errors in evaluation of force encoding in neuronal discharges, an effect that can be detected by monitoring forces and force dynamics.

### Contribution of “Creep” to Discharges to Decreasing Forces

The finding that discharges to force decreases are prolonged after sustained leg bending implied that sensory encoding was time and history dependent. We examined the effects of stimulus duration on cuticle properties and sensory discharges by imposing ramp and hold functions (constant bending force mode) at the same rates of rise and decline but varying the duration of the hold phase (Fig. 7*A*). Measurements of displacement in these tests showed gradual increases due to “creep,” which in viscoelastic materials is related to the duration of constant force application (Fig. 7*B*). Measurements of the discharge frequencies of 6A receptors to force decreases showed that they increased following prolonged hold phases (Fig. 7*A*), as was found for cutaneous mechanoreceptors of vertebrates (Ref. 50). Furthermore, the increased discharge frequency was correlated with an increase in the displacement resulting from mechanical creep in the cuticle (Fig. 7*C*). These data support the idea that discharges to force decreases result, in part, from mechanical properties of the exoskeleton.

### Similarities to Viscoelastic Mechanisms in Other Sensory Receptors

Campaniform sensilla sense forces through the exoskeleton, which acts as both a structural element for muscle actions and force generation and as the “skin” of the insect. Clear parallels with the results of the present study can be found in previous investigations on cutaneous receptors of vertebrates. Sense organs of the skin can show discharges to both stimulus increases and decreases (on-off responses) that are highly sensitive to and encode the rate of force application and release of skin deformation (humans: Refs. 41 and 50; cats, Ref. 51; raccoons, Ref. 52; squirrel monkeys, Ref. 53). Viscoelasticity of the capsule surrounding the endings and the adjacent skin is considered to play a major role in the generation of the responses to force decreases (off-responses). In responses to ramp and hold stimuli the firing frequencies during “off” discharges of cutaneous rapidly adapting afferents shows a strong dependence on immediately preceding plateau duration and were greatly reduced or absent with short plateau durations (54), an effect that was attributed to the time needed for “viscous pressures” to develop. Furthermore, Pubols (53) suggested that “regional and species differences in functional properties of cutaneous mechanoreceptors are more likely attributable to differences in mechanical properties of skin and subadjacent tissues than to any inherent differences in receptor properties.”

Experiments and modeling of sensory discharges in Pacinian corpuscles, which also discharge to stimulus increases and decreases, provide additional insights into the effects of viscoelasticity on the generation of sensory responses to force decreases (40, 55, 56). The sensory nerve ending in the Pacinian corpuscle is surrounded by a lamellated, fluid-filled capsule that acts as a viscoelastic coupling mechanism (57). Characteristics of viscoelasticity are, therefore, not only present in the surrounding skin but inherent in the capsule itself. The relatively large size of the capsule allows for experimental manipulation and several studies have shown that sensory discharges to force decreases are eliminated after removal of the outer lamellae of the capsule. In describing the mechanism underlying this effect, Loewenstein and Skalak (55) proposed that when the corpuscle is released from compression, energy stored in the elastic elements during compression is released producing viscous flow in the capsule, whose magnitude depends upon the velocity of release. The resultant difference in “viscous pressure” acts on the sensory dendrite to activate mechano-gated ion channels, and produce generator potentials (56, 58) that are generally similar (or slightly smaller) in magnitude and time course as those occurring during compression of the sense organ. Thus, “off” and “on” responses produce similar effects on the nerve ending. It is important to note that, in contrast to Pacinian corpuscles that are radially symmetric, the caps of campaniform sensilla are asymmetric (17) and different sensory neurons in a group are excited by force increases or decreases due to the mechanical directional filtering provided by the caps.

### Summary: mechanisms Underlying Responses to Decreasing Forces

**Figure 10.**
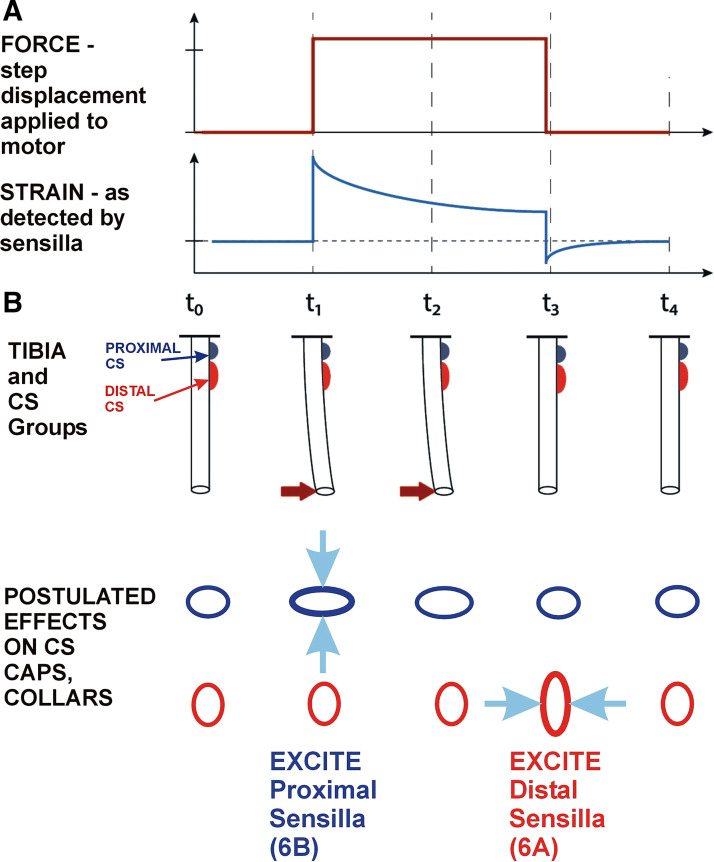
Summary: mechanisms underlying responses to decreasing forces. *A*: forces and strains—force are applied as a step displacement, hold and decrease on the end of the tibia. Discharges of campaniform sensilla (CS) indicate that the strains increase then decline during the hold phase. Force decreases produce strains similar to force increases in the opposite direction. *B*: graphical model—force increases initially produce compressive strains that activate the proximal (6B) sensilla. During the hold phase, viscoelasticity in the cuticle generates “creep” and transiently acts to create a new “neutral” position. Decrease in forces produces strains similar to force increases in the opposite direction transiently activating the distal (6A) receptors.

Figure 10 presents schematic diagrams of the leg and tibial campaniform sensilla that summarize the results of this study. A bending force in the direction of extension is applied at *t* = 1 to the cockroach tibia on its distal end. The force is then held for one second and released at *t* = 2. The bending force initially produces elastic deformation, generating compressive strains on the side opposite the point of force application that result in a discharge of the proximal tibial sensilla, whose caps are perpendicular to the long axis of the tibia. However, cuticle is both elastic and viscoelastic. The tibia is stiff to rapidly applied forces but that resistance decreases over time (stress relaxation) due to viscoelasticity in the cuticle. During this time the tibia undergoes slight change in its shape and the magnitude of the viscous deformation (creep) depends upon the duration of force application. The cuticle of the tibia, therefore, functionally approaches a new “neutral” position. Release from the bending force results in “unbending” and return of the tibial cuticle to its initial shape. Transiently, this generates compressive strains parallel to the long axis of the tibia, acting like a force in the opposite direction (flexion) and producing discharge in the distal sensilla, whose caps are parallel to the tibial axis. The processes of transient deformation and restoration of shape are reversible. Similar processes occur in the stick insect tibia with Group 6B excited by imposed force and Group 6A activated upon force release.

The effects of offset loads on the cuticle may be also mechanical. In large imposed offset forces, it is possible that the elastic mechanical distortion of the cuticle exceeds the magnitude of shape changes by viscoelastic properties and the effects of force decreases never reach the threshold needed for activation of the 6A or distal sensilla upon partial release of forces. To smaller offsets, CS discharges still occur as phasicotonic firing at lower frequencies that resembles sensory responses to forces in the opposite direction (Figs. 4 and 5). Preliminary observations indicate that these responses are rate sensitive although this has not been systematically examined. Dickinson (44) studied the effects of offsets upon discharges of CS of the wings of blow flies, which respond bidirectionally. That study showed that offset forces strongly affected polarity of the deflections to which the neurons responded and receptors only responded to stimuli that increased the deflection of the wing and not to those that moved the wing toward the undeflected position.

It is important to note that this schematic formulation does not distinguish between the properties of structures immediately involved in mechanotransduction (linking the sensory dendrite to the cuticular caps) and the properties of the cuticle of the leg. The cap/collar itself has been shown to be viscoelastic in some sensilla while about one-third were purely elastic (59) but its potential functional effects have not been determined (see Ref. 44). In addition, the cuticle immediately surrounding some groups of campaniform appears morphologically specialized (devoid of pore canals) and can show fluorescence that is higher than the remaining exoskeleton (Ref. 30, Fig. 2) (60). The measurements of Chapman et al. (33, 34) support the idea that this specialized cuticle may play a significant role in determining viscoelasticity properties of sensory discharges but further experiments are needed.

### Mathematical Model of Campaniform Sensilla Reproduces Characteristics of Responses to Decreasing Forces

We have developed a mathematical model of force encoding by campaniform sensilla (25, 37). The model calculates the discharge frequency of the receptors as the sum of an adaptive function (the instantaneous force, minus the force as processed by a low-pass filter) and a function reflecting tonic sensitivity (adjusted by an offset). Characteristics of the campaniform sensilla, such as rate sensitivity and hysteresis, are not specifically modeled but, instead, are emergent properties of the model, as confirmed by tests using standard ramp and hold waveforms (25). The present study shows that the model also accurately replicates the effects of offset forces on sensory discharges, even though this phenomenon was not considered while developing the model. Interestingly, because the model naturally produces off responses but indenting CS caps direction does not, the model presumably incorporates the “creep” of the cuticle in response to loading. Future work is necessary to determine what proportion of the model’s adaptation represents changes in mechanical forces versus properties of mechanotransduction in the sensory cells.

What insight does the model’s behavior provide about the mechanisms underlying “off” responses? Recall that the model’s discharge contains an adaptive component, which is proportional to the applied force at that instant, minus the dynamic threshold variable. The dynamic threshold variable follows the applied force, such that if a force is applied for a long time, the threshold reaches the value of the applied force, and the resulting discharge is 0. Then, when the direction of the force changes suddenly, e.g., when a negative force stimulus is removed, the threshold initially remains near the stimulus force level, contributing to high-amplitude discharge. The model’s behavior is consistent with the phenomena discussed in Fig. 9. The model provides a system in which ideas for experiments may be tested before complicated and time-consuming experimental work.

How does “creep” affect the model discharge? When a force stimulus is applied to the model, its dynamic threshold changes very slowly, never reaching the value of the force over the duration of the stimulus. As a result, the threshold continues to “creep” the longer a force stimulus is applied, mimicking the “creep” measured in the insect cuticle. This creep serves to amplify the effect described in the previous paragraph, because the “off” responses depend on the level of the dynamic threshold, and a longer stimulus causes a greater change in the threshold’s value. In this way, the amplitude of the model’s “off” responses reflect the duration of the stimulus as observed in animal experiments. However, it should be noted that the degree of “creep” in the model is not as severe as that measured in the animals. In future work, we plan to expand the model to explicitly consider the properties of the cuticle separately from the transduction mechanism. Such a model could enable one to predict the sensory discharges experienced by an animal based on measurements of the cuticle stiffness. The resulting work could shed light into how sensory feedback properties may depend on the time since an insect’s last molt.

### Limitations to This Study

Our use of the Aurora system, which precisely controlled both force and displacement, indicated that there was considerable variability in the compliance of the cuticle in the legs of stick insects used in experiments. The cuticle becomes increasingly sclerotized in the time after the adult molt and this time period was not monitored or controlled in our studies. In addition, we did not measure the elastic modulus directly and previous studies have indicated that its value may vary as a gradient in the leg cuticle (31, 32). Our measurement of compliance through probe displacement therefore includes diverse regions, including the shaft of the tibia, the areas surrounding the campaniform sensilla, and proximal regions near the femoro-tibial joint (which was immobilized). Studies are planned to more carefully examine the mechanical properties of sections of tibia by both bending and axial tensions and compression (to avoid the limitations of measures of indentation hardness) (61). Those experiments could also potentially permit correlation of the weight and age of the animal with the extent of cuticle sclerotization (which have not been monitored in the present studies). Measurements of cuticle properties would also permit more precise quantification of the differences we observed in the responses of campaniform sensilla in stick insects and cockroaches. It should be noted, that potential variability in mechanical properties has not been addressed in many previous studies that used displacement as a controlled variable, without directly monitoring the resultant forces.

### Decision-Making Based upon Tuned Proprioceptive Feedback: Advantages and Functions of Discharges to Decreasing Forces

Our studies support the idea that the response properties of tibial campaniform sensilla are adaptively tuned by properties of the cuticle and mechanisms of mechanotransduction, to provide active signals of large or rapid force decreases as occur prior the onset of swing or loss of substrate engagement. Many sense organs are tuned to detect the ranges and properties of behaviorally relevant stimuli (62). The specific tuning of campaniform sensilla to the dynamics of decreasing forces is also computationally efficient as it provides rapid, discrete signals of force decrements, which otherwise would require detection by processing of signals of force levels (3, 63, 64). The generation of these signals through viscoelastic properties of the cuticle implies that they may vary depending upon the age of the animal and this will be examined in future studies.

Although similar receptors are found in other invertebrates, vertebrate Golgi tendon organs have been shown to encode only force increases, not decreases. Detection of load variations through monitoring of discharges to force levels and increases (+d*F*/d*t*) is implied by previous findings that small changes imposed upon muscle tendons can modulate muscle active in support and propulsion (14). However, vertebrates also can detect force decrements through other receptors, such as cutaneous receptors of the feet (41) or vestibular organs, which have been postulated to play a significant role in the detections of postural perturbations. Integration of multimodal signals is thought to occur in determination of the timing of onset of swing in both vertebrates and invertebrates. The use of local, “dedicated” signals in insects could make the process faster and may also permit higher rates of stepping. The computational efficiency and simplification these signals provide could also be applied in control of walking machines to similar advantage.

## GRANTS

This study was supported by National Science Foundation (NSF Collaborative Research in Computational Neuroscience) CRCNS Grant 2113028 (to N.S.S. and S.N.Z.), DFG (Deutsche Forschungsgemeinschaft) SFB 1451 (project-ID 431549029-A05) (to A.B.), NSF DBI 2015317 as part of the Next Generation Networks for Neuroscience Program (to N.S.S.), and NSF Grant MRI 0959012.

## DISCLOSURES

No conflicts of interest, financial or otherwise, are declared by the authors.

Ansgar Büschges is an editor of *Journal of Neurophysiology* and was not involved and did not have access to information regarding the peer-review process or final disposition of this article. An alternate editor oversaw the peer-review and decision-making process for this article.

## AUTHOR CONTRIBUTIONS

C.M.H., N.S.S., and S.N.Z. conceived and designed research; C.M.H., N.S.S., and S.N.Z. performed experiments; C.M.H., N.S.S., and S.N.Z. analyzed data; N.S.S., A.B., and S.N.Z. interpreted results of experiments; C.M.H., N.S.S., and S.N.Z. prepared figures; C.M.H., N.S.S., and S.N.Z. drafted manuscript; N.S.S., A.B., and S.N.Z. edited and revised manuscript; C.M.H., N.S.S., A.B., and S.N.Z. approved final version of manuscript.
